# The cryptic plasmid is more important for *Chlamydia muridarum* to colonize the mouse gastrointestinal tract than to infect the genital tract

**DOI:** 10.1371/journal.pone.0177691

**Published:** 2017-05-25

**Authors:** Lili Shao, Jose Melero, Nu Zhang, Bernard Arulanandam, Joel Baseman, Quanzhong Liu, Guangming Zhong

**Affiliations:** 1 Department of Microbiology and Immunology, University of Texas Health Science Center at San Antonio, San Antonio, Texas, United States of America; 2 Department of Dermatovenereology, Tianjin Medical University General Hospital, Tianjin, China; 3 Department of Biology, University of Texas at San Antonio, San Antonio, Texas, United States of America; Universita degli Studi di Bologna, ITALY

## Abstract

*Chlamydia* has been detected in the gastrointestinal tracts of both animals and humans. However, the mechanism by which *Chlamydia* colonizes the gut remains unclear. *Chlamydia muridarum* is known to spread from the genital to the gastrointestinal tracts hematogenously. The *C*. *muridarum* plasmid is a key pathogenic determinant in the mouse upper genital tract although plasmid-deficient *C*. *muridarum* is still able to colonize the upper genital tract. We now report that plasmid-deficient *C*. *muridarum* exhibits significantly delayed/reduced spreading from the mouse genital to the gastrointestinal tracts. *C*. *muridarum* with or without plasmid maintained similar levels in the mouse circulatory system following intravenous inoculation but the hematogenous plasmid-deficient *C*. *muridarum* was significantly less efficient in colonizing the gastrointestinal tract. Consistently, plasmid-deficient *C*. *muridarum* failed to restore normal colonization in the gastrointestinal tract even after intragastric inoculation at a high dose. Thus, we have demonstrated a plasmid-dependent colonization of *C*. *muridarum* in the gastrointestinal tract, supporting the concept that *C*. *muridarum* may have acquired the plasmid for adaptation to the mouse gastrointestinal tract during oral-fecal transmission. Since the plasmid is more important for *C*. *muridarum* to colonize the gastrointestinal tract than to infect the genital tract, the current study has laid a foundation for further defining the host pathways targeted by the plasmid-encoded or -regulated chlamydial effectors.

## Introduction

*Chlamydia trachomatis* is a leading cause of sexually transmitted bacterial infection, which, if not treated appropriately, can lead to tubal adhesion/hydrosalpinx/infertility in women [1–3]. However, the mechanisms by which *C*. *trachomatis* causes upper genital tract pathology remain unclear. *Chlamydia muridarum* infection in the mouse genital tract can cause hydrosalpinx and infertility [4–6], which has been used for investigating the mechanisms of *C*. *trachomatis* pathogenesis [7–12]. It is worth noting that there are clear differences between the *C*. *muridarum-*mouse model and *C*. *trachomatis* infection in humans. For example, a single intravaginal infection with *C*. *muridarum* can induce hydrosalpinx at a rate of >50% in many strains of mice [6] while it is thought to take repeated infections with *C*. *trachomatis* to cause fallopian tubal adhesion/tubal infertility in a portion of women [1–3]. Nevertheless, the robust murine model has allowed us to reveal both chlamydial [13–17] and host [7, 11, 18–24] factors involved in *C*. *muridarum* induction of hydrosalpinx [6, 16, 25, 26], which has provided new insights into the mechanisms of chlamydial pathogenicity. For example, the cryptic plasmid has been shown to be a critical determinant for *C*. *muridarum* pathogenicity in the mouse upper genital tract [13, 16, 17, 27, 28].

Like most *Chlamydia* species, the *C*. *muridarum* plasmid, termed pMoPn/pCM [29, 30], codes 8 putative proteins designated as plasmid-encoded glycoproteins 1 to 8 (pGP1-8) [28, 29, 31]. Some pGPs, such as pGP3 [13, 32], may directly exert virulence functions while others, such as pGP4 [33, 34] and pGP5 [35], may regulate other chlamydial genes, including the chromosomal genes involved in glycogen synthesis. Plasmid-deficient *C*. *muridarum* no longer induced significant hydrosalpinx following intravaginal inoculation [16, 17]. The lack of hydrosalpinx induction correlated with both reduced tubal inflammation and decreased ascending infection. Furthermore, following transcervical inoculation to bypass the cervical barrier, plasmid-deficient *C*. *muridarum* was enabled to induce hydrosalpinx in some strains of mice including CBA/1J [36], demonstrating a critical role of the plasmid in promoting *C*. *muridarum* to cross the cervical barrier. This also suggested that plasmid-deficient *C*. *muridarum* organisms still possess the ability to infect the upper genital tract, invade the oviduct and induce hydrosalpinx-causing tubal inflammation. Nevertheless, when vaginal shedding of live organisms was compared between mice infected with plasmid-competent versus -deficient *C*. *muridarum*, there was no significant difference [13, 16, 17, 27, 36], suggesting that the plasmid is not essential for *C*. *muridarum* infection in the mouse genital tract, especially the lower genital tract. Plasmid-free *C*. *trachomatis* have been isolated from human urogenital tracts [37, 38] although the plasmid could have been lost during the *in vitro* recovery process. More importantly, it was recently reported that *C*. *trachomatis* serovar D organisms with or without plasmid achieved similar levels of infection in the genital tracts of nonhuman primates [39]. These observations suggest that the plasmid may not always be essential for *C*. *trachomatis* to infect the genital tract.

Although *C*. *trachomatis* is a sexually transmitted pathogen known to infect the genital tracts of humans [40, 41], it has been frequently detected in the human gastrointestinal (GI) tracts [42–45]. Interestingly, when *C*. *muridarum* was introduced to multiple mouse mucosae, it readily colonized the GI tract [46]. We have recently shown that genital tract *C*. *muridarum* spread to and established long-lasting colonization in the GI tract [47], confirming previous findings on *C*. *muridarum* colonization of the GI tract for long periods of time [48, 49]. Following a single intravaginal inoculation with wild type *C*. *muridarum*, the *C*. *muridarum* organisms were cleared from the genital tract in 4 to 5 weeks but the organisms spread to the GI tract persisted and the genital tract hydrosalpinx remained in the same mice for long periods of time [47], suggesting a time correlation between the GI tract *C*. *muridarum* and the genital tract pathology. However, it is not clear whether and how the GI tract *C*. *muridarum* contributes to the genital tract pathology at this moment. Interestingly, the genital tract *C*. *muridarum* spreading into the GI tract of the same mouse is likely independent of the oral-fecal route since singly housed mice restrained from taking up mouse secretions in a net-bottom cage still allowed *C*. *muridarum* to spread from the genital to the GI tracts [46, 47]. The spreading has been shown to be via a hematogenous route since *C*. *muridarum* survived in the blood and the hematogenous *C*. *muridarum* established long-lasting colonization restricted to the GI tract [50]. However, the GI tract *C*. *muridarum* failed to auto-inoculate the genital tract even after colonization in the GI tract for 70 days [51], which is inconsistent with the popular belief that the GI tract *C*. *muridarum* organisms may serve as a reservoir for auto-inoculation of the genital tract [49, 52]. Thus, if the GI tract *C*. *muridarum* can affect the genital tract pathology at all, it may be via an indirect mechanism but not by directly autoinoculating the genital tract. Regardless of how GI tract *C*. *muridarum* may affect the genital tract pathology, the above observations/analyses together suggest that *C*. *muridarum* is well adapted to the GI tract environment. Thus, we hypothesize that *C*. *muridarum* may have acquired the plasmid for improving its colonization in the GI tract. Although *C*. *muridarum* colonization in the GI tract may be harmless, testing our hypothesis by evaluating the role of chlamydial factors in *C*. *muridarum* colonization in the GI tract is biologically significant. This is because the same “virulence factors” *C*. *muridarum* has acquired to improve its fitness in the GI tract may enable *C*. *muridarum* to infect extra-GI tract tissues, such as overcoming the cervical barrier in the genital tract [36]. Thus, defining the molecular basis of *C*. *muridarum* interactions with the GI tract will help us to understand the *C*. *muridarum* pathogenic mechanisms in the genital tract.

In the current study, we tested the above hypothesis and found that plasmid-deficient *C*. *muridarum* exhibited significantly delayed/reduced spreading from the mouse genital to the GI tracts although plasmid-deficiency did not significantly alter *C*. *muridarum* live organism shedding in the genital tracts. The reduced spreading of plasmid-deficient *C*. *muridarum* to the GI tract was due to inefficient colonization of plasmid-deficient *C*. *muridarum* in the GI tract since plasmid-deficient *C*. *muridarum* significantly decreased colonization in the GI tract following intragastric inoculation. The current studies have demonstrated a significant role of the plasmid in promoting *C*. *muridarum* colonization in the GI tract. Since the plasmid impact on *C*. *muridarum* infection in the genital tract is not as obvious [13, 16, 17, 27, 36], our current finding not only suggests that *C*. *muridarum* may have acquired the plasmid for adaptation to the GI tract but also provides a foundation for further defining the host pathways targeted by the plasmid-encoded or regulated effectors. The same molecular mechanisms evolved during *C*. *muridarum* adaptation to the GI tract mucosal tissue might also be used by *C*. *muridarum* to induce hydrosalpinx when *C*. *muridarum* is artificially introduced into the genital tract mucosal tissue.

## Materials and methods

### *Chlamydia muridarum* and mouse infection

*Chlamydia muridarum* strain Nigg organisms (initially acquired from Dr. Robert C. Brunham’s lab at the University of Manitoba in 1999) were propagated and purified in HeLa cells (human cervical carcinoma epithelial cells, ATCC cat# CCL2) as described previously [47, 53]. After comparing its full genome sequence (Genbank accession# CP009760.1) with that of the reference *C*. *muridarum* [30], we re-designated this strain as Nigg3. A plasmid-deficient clone (designated as CMUT3.G5) was derived from Nigg3 as described previously [13, 35]. The pGFP:CM plasmid [54] was transformed into the plasmid-deficient CMUT3.G5 clone to produce plasmid-competent (designated as CMUT3G5-pGFP or CM-pGFP) *C*. *muridarum* organisms as described previously [13, 35]. The above *C*. *muridarum* organisms were propagated and purified as elementary bodies (EBs). Aliquots of the purified EBs were stored at -80oC until use.

Six to seven week-old female C57BL/6J (stock number 000664) and CBA/J (000656, both from Jackson Laboratories, Inc., Bar Harbor, Maine) mice were inoculated with purified *C*. *muridarum* EBs at between 1 X 10^4^ to 1 X 10^7^ inclusion-forming units (IFUs) via different routes as indicated in individual experiments. After inoculation, mice were monitored for vaginal and rectal live organism shedding and bled for titrating chlamydial genomes in the blood. Mice with the same treatment were housed in the same groups in the same cage. In our pilot experiments, we did comparison between singly housing and group housing [47, 50]. We found that hematogenous spreading from the genital to the GI tract is the dominant route for *C*. *muridarum* to reach the GI. The results were similar between mice singly housed and those housed in groups in the same cage. Thus, to save research funds, we have used group housing in most experiments in the current study.

The animal experiments were carried out in accordance with the recommendations in the Guide for the Care and Use of Laboratory Animals of the National Institutes of Health. The protocol was approved by the Committee on the Ethics of Laboratory Animal Experiments of the University of Texas Health Science Center at San Antonio.

### Titrating live chlamydial organisms recovered from swabs

For monitoring live organism shedding, vaginal and rectal swabs were taken on day 3 post-inoculation and weekly thereafter. To quantitate live chlamydial organisms, each swab was soaked in 0.5 ml of SPG and vortexed with glass beads. The chlamydial organisms released into the supernatants were titrated on HeLa cell monolayers in duplicate. The infected cultures were processed for immunofluorescence assay as described previously [16, 26]. Inclusions were counted in five random fields per coverslip under a fluorescence microscope. For coverslips with less than one IFU per field, entire coverslips were counted. Coverslips showing obvious cytotoxicity of HeLa cells were excluded. The total number of IFUs per swab was calculated based on the mean IFUs per view, the ratio of the view area to that of the well, dilution factor, and inoculation volumes. Where possible, a mean IFU/swab was derived from the serially diluted samples for any given swab. The total number of IFUs/swab was converted into log_10_ and used to calculate the mean and standard deviation across mice of the same group at each time point.

### Titrating the number of *C*. *muridarum* genomes in the mouse blood using quantitative PCR

To quantitate the genome copies of *C*. *muridarum*, 20μl of fresh blood was transferred to the lysis buffer provided with Quick-gDNA miniPrep Kit (Cat#: 11-317C, Genesee Scientific, San Diego, CA) and subjected to DNA extraction following manufacturer’s instruction. Each DNA preparation was eluted in 100μl elution buffer, and 2μl was used for quantitative PCR (qPCR). The following primers derived from the chlamydial 16S rRNA coding region were used: forward primer (5′-CGCCTGAGGAGTACACTCGC-3AGGA), reverse primer (5′-CCAACACCTCACGGCACGAG-3‘) and Double-Quenched Probe (5′-CACAAGCAGTGGAGCATGTGGTTTAA-3′) (Integrated DNA Technologies, Coralville, Iowa). PCR was carried out in a total volume of 10 μ in a CFX96 Touch Deep Well Real-Time PCR Detection System with IQ Supermix real-time PCR reagent (Bio-Rad, Hercules, CA). Genome copy numbers for a given sample in triplicate were calculated based on a standard plasmid DNA prep in the corresponding samples and expressed as Log_10_ genomes per 10ul blood. The qPCR conditions included an initial denaturation step at 95°C for 3 minutes, followed by 40 cycles of amplification at 95°C for 15 seconds and 60°C for 1 minute.

### Statistics analyses

All data including the time courses for the number of live organisms (IFUs), genome copies and infection rates were compared using “area-under-the-curve or AUC” or “days to clearance” between two groups of mice using Wilcoxon rank sum test. For AUC calculation from the IFU data, total number of Log_10_ IFUs collected from vaginal or rectal swabs of a given mouse over the entire time course was used to represent the AUC for that mouse for the purpose of comparison between the plasmid-deficient and -competent *C*. *muridarum*-infected groups. For AUC calculation from the % of mice positive for IFU data, the % of mice positive for IFU at each time point was used as an independent AUC variable in a given group. Thus, the number of the time points observed in a given experiment determined the group sample size for the purpose of comparison between groups. In order to compare the days to clearance between the plasmid-deficient and -competent *C*. *muridarum*-infected groups, the number of days for a given mouse to remain positive for IFUs was used as the “days to clearance” for that mouse.

## Results

### Plasmid-deficient *C*. *muridarum* is significantly attenuated in spreading to the mouse GI tract

**Fig 1:**
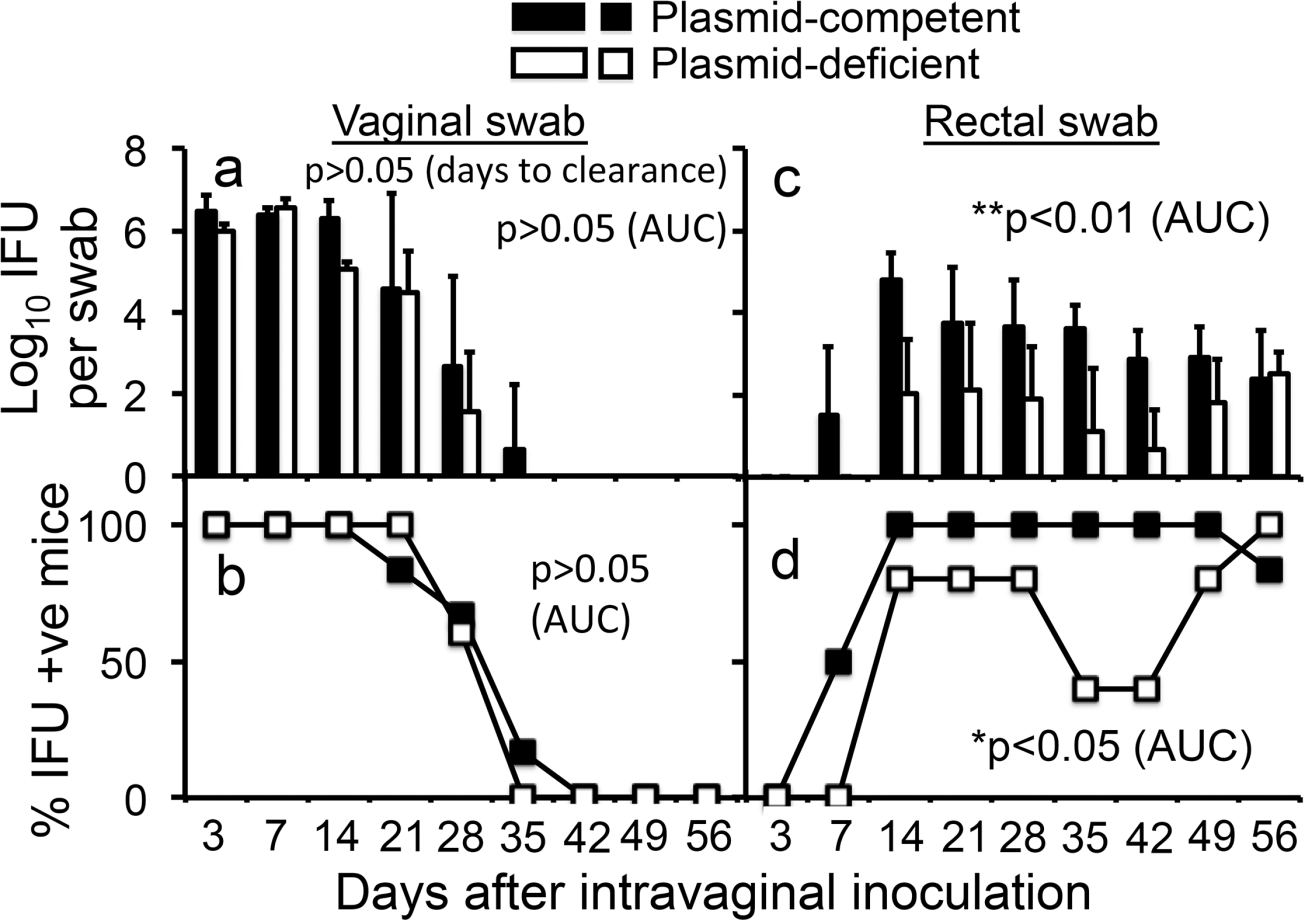
Comparing *C*. *muridarum* with or without plasmid for their ability to spread to the GI tracts of C57BL/6J mice following an intravaginal inoculation. The plasmid-competent (Filled bar or square; n = 6) or -deficient (open bar or square; n = 5) *C*. *muridarum* organisms were intravaginally inoculated into female C57BL/6J mice with 2 x 10^5^ inclusion forming units (IFUs) per mouse. At various time points post inoculation as indicated along the X-axis, both vaginal (panels a & b) and rectal (c & d) swabs were taken for titrating live organisms and the recovered live organisms were expressed as Log_10_ IFUs per swab as displayed along the Y-axis (panels a & c) and % of mice detected positive for IFUs at a given time point were plotted in panels b & d. The data were collected from two independent experiments. Note that both plasmid-deficient and -competent *C*. *muridarum* displayed similar live organism shedding courses from the genital tract (panels a & b, p>0.05, area under the curve or days to clearance, Wilcoxon rank sum). However, plasmid-deficient *C*. *muridarum* developed a significantly delayed/reduced shedding course from the same mouse GI tracts in terms of both the level of shedding (panel c, **p<0.01, area under the curve, Wilcoxon rank sum) and number of mice remaining positive for shedding (panel d, *p<0.05, area under the curve, Wilcoxon rank sum).

We have previously shown that *C*. *muridarum* can effectively spread from the mouse genital to the GI tracts [47]. In the current study, we evaluated whether the chlamydial spreading to the GI tract is dependent on the cryptic plasmid by comparing plasmid-competent and -deficient *C*. *muridarum* for colonizing the GI tract following an intravaginal inoculation. When tested in C57BL/6J mice (Fig 1), we found that both plasmid-competent and -deficient *C*. *muridarum* displayed a similar live organism shedding course from the lower genital tract, confirming the previous findings [13, 16, 17, 27]. However, plasmid-deficient *C*. *muridarum* exhibited significantly decreased live organism shedding in the rectal swabs taken from the same mice. Live organisms were detected in the rectal swabs of mice infected with plasmid-competent *C*. *muridarum* as early as day 7 after intravaginal inoculation but delayed until day 14 in the mice similarly infected with plasmid-deficient *C*. *muridarum*. Furthermore, plasmid-deficient *C*. *muridarum* developed significantly lower titers of live organisms at most time points. Only by day 56 after intravaginal inoculation, did the titers of plasmid-competent and -deficient *C*. *muridarum* reach similar levels. Overall, by comparing the area under the curve, mice infected with plasmid-deficient *C*. *muridarum* developed significantly reduced live organism shedding in terms of both the infectious titers and infection frequencies, demonstrating an important role of the plasmid in *C*. *muridarum* spreading to the GI tract. However, the live organism shedding from the vaginal swabs was similar between mice infected with *C*. *muridarum* with or without plasmid, which is consistent with various previous reports that the plasmid is not essential for *C*. *muridarum* to infect the mouse genital tract [13, 16, 17, 27, 36].

**Fig 2:**
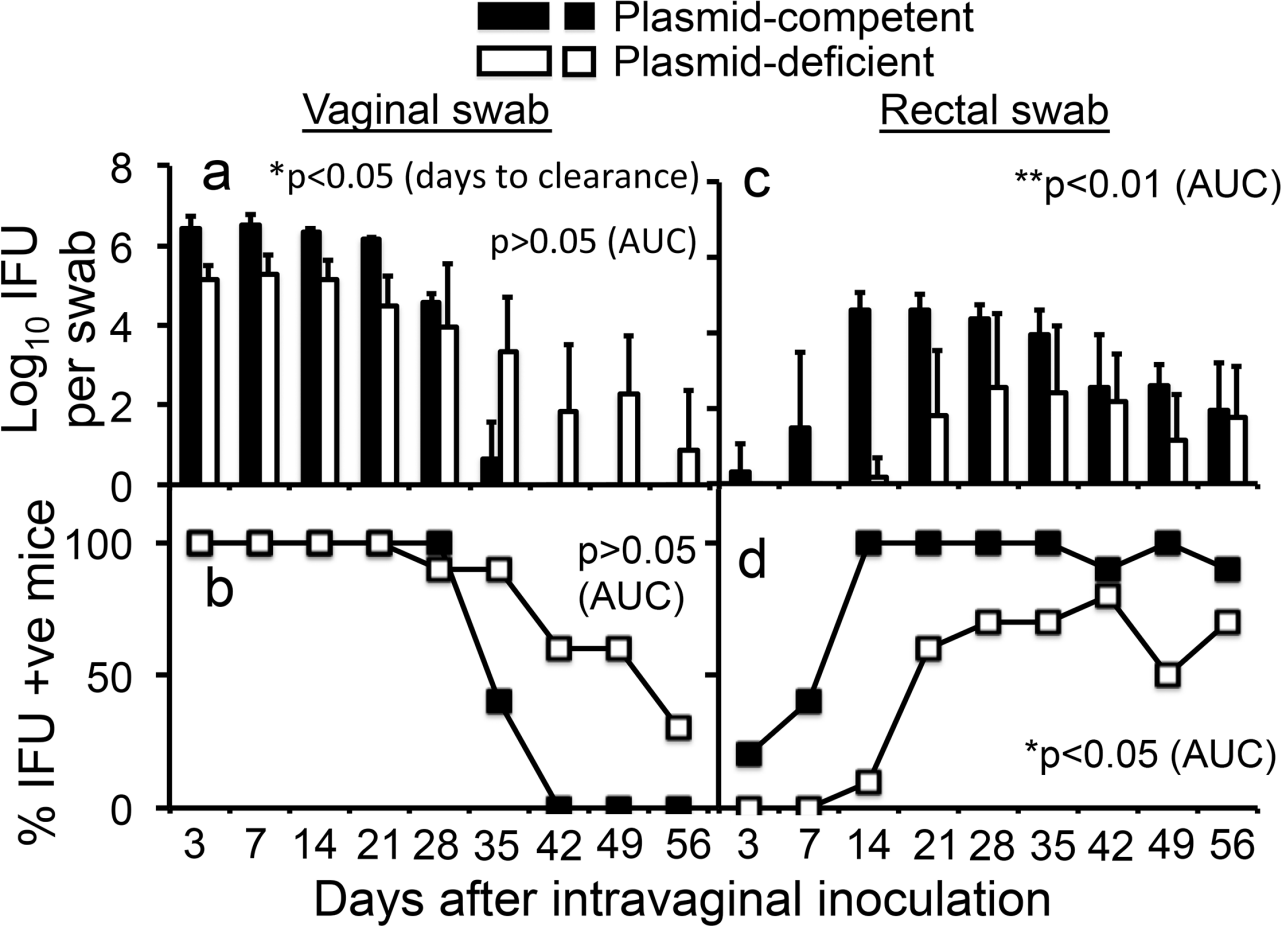
Comparing *C*. *muridarum* with or without plasmid for their ability to spread to the GI tracts of CBA/1J mice following an intravaginal inoculation. The experiments were carried out and the results were presented as described in Fig 1 legend above. The plasmid-competent *C*. *muridarum* organisms were inoculated into 5 while the plasmid-deficient to 10 mice. The data were collected from 2 independent experiments. Plasmid-deficient *C*. *muridarum* displayed significantly longer shedding from the mouse genital tracts (panel a, p<0.05, days to clearance, Wilcoxon rank sum) but the overall shedding courses were similar between plasmid-deficient and -competent *C*. *muridarum* (panels a & b, p>0.05, area under the curve, Wilcoxon rank sum). However, plasmid-deficient *C*. *muridarum* developed significantly delayed/reduced shedding courses in the GI tracts in terms of both the level of shedding (panel c, **p<0.01, area under the curve, Wilcoxon rank sum) and number of mice remaining positive for shedding (panel d, *p<0.05, areas under the curve, Wilcoxon rank sum).

We further compared the plasmid-competent and -deficient *C*. *muridarum* organisms in CBA/1J mice since this strain is more susceptible to *C*. *muridarum* infection [6]. As shown in Fig 2, plasmid-deficient *C*. *muridarum* developed lower levels of live organism shedding from the vaginal swabs but with a significantly prolonged shedding course (when days to clearance were compared). The overall vaginal shedding courses between plasmid-competent and -deficient *C*. *muridarum* were not significantly different (when AUC was compared). Despite the prolonged shedding course of plasmid-deficient *C*. *muridarum* in the CBA mouse genital tract, the plasmid-deficient *C*. *muridarum* organisms developed significantly delayed/reduced shedding courses from the rectal swabs. Plasmid-competent *C*. *muridarum* started to appear in the rectal swabs as early as day 3 but plasmid-deficient *C*. *muridarum* only became detectable on day 14. The titers of plasmid-deficient *C*. *muridarum* were significantly lower than those of plasmid-competent *C*. *muridarum* in all rectal swabs except for the one taken on day 56.

### The decreased spreading into the mouse GI tract by plasmid-free *C*. *muridarum* is unlikely due to lack of survival in the blood

**Fig 3:**
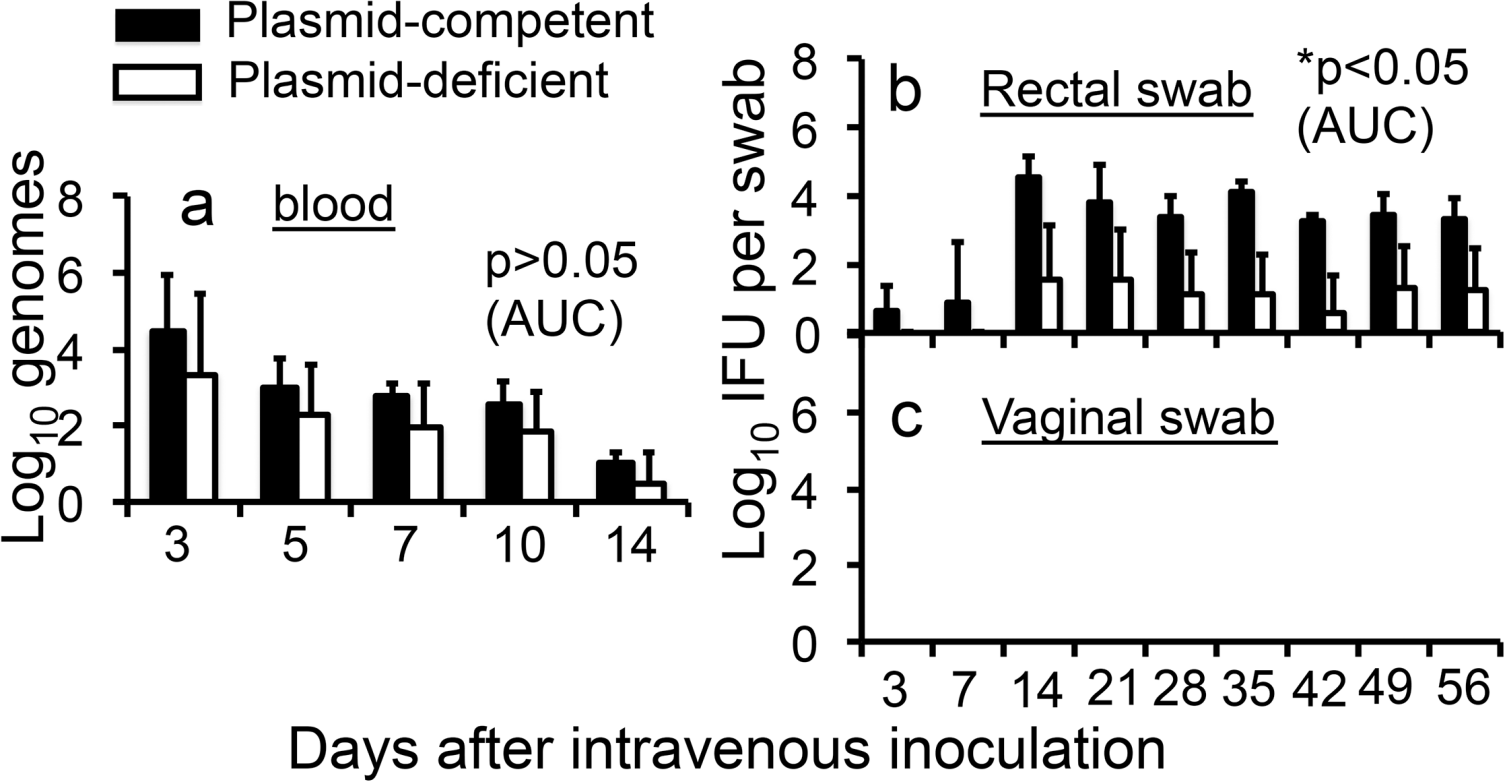
Comparing *C*. *muridarum* with or without plasmid for their ability to spread to the GI tracts of C57BL/6J mice following an intravenous inoculation. The plasmid-competent (solid bar; n = 4) or -deficient (open bar; n = 7) *C*. *muridarum* organisms were inoculated into female C57BL/6J mice with 2 x 10^6^ IFUs per mouse via a retro-orbital injection. At various time points post inoculation as indicated along the X-axis, blood drops (panel a), rectal (b) and vaginal (c) swabs were taken for quantitating *C*. *muridarum* genomes or titrating live organisms. The recovered genome copies and live organisms were expressed as Log_10_ genomes per 10μl blood (a) or Log_10_ IFUs per swab (b & c) as displayed along the Y-axis. The data were obtained from two experiments. Note that although both plasmid-competent and -deficient *C*. *muridarum* displayed similar genome recovery time courses from the blood (panel a, p>0.05, area under the curve, Wilcoxon rank sum), plasmid-deficient *C*. *muridarum* developed a significantly delayed/reduced shedding course from the GI tracts (b, *p<0.05, areas under the curves, Wilcoxon rank sum). No live organisms were detected in the genital tracts of any mice (c).

We have previously shown that *C*. *muridarum* can spread from the mouse genital tract to GI tract hematogenously [50]. We then tested whether the decreased spreading to the GI tract by plasmid-deficient *C*. *muridarum* was due to the reduced survival in the blood. To avoid contamination by the input organisms, we used a retro-orbital injection to deliver *C*. *muridarum* organisms into the blood circulation system and monitored the blood levels of *C*. *muridarum* by collecting blood from the tail vein. As shown in Fig 3, there was no significant difference in genome recovery time courses between plasmid-competent and -deficient *C*. *muridarum*. Furthermore, consistent with what we have previously shown [50], the hematogenous plasmid-competent *C*. *muridarum* developed a long-lasting colonization in the GI tract but without any significant spreading to the genital tract (Fig 3C). However, plasmid-deficient *C*. *muridarum* was less efficient in spreading to and colonizing the GI tracts from the blood circulatory system (Fig 3B).

### Plasmid-deficient *C*. *muridarum* is attenuated in colonizing the mouse gastrointestinal tracts following intragastric inoculation

**Fig 4:**
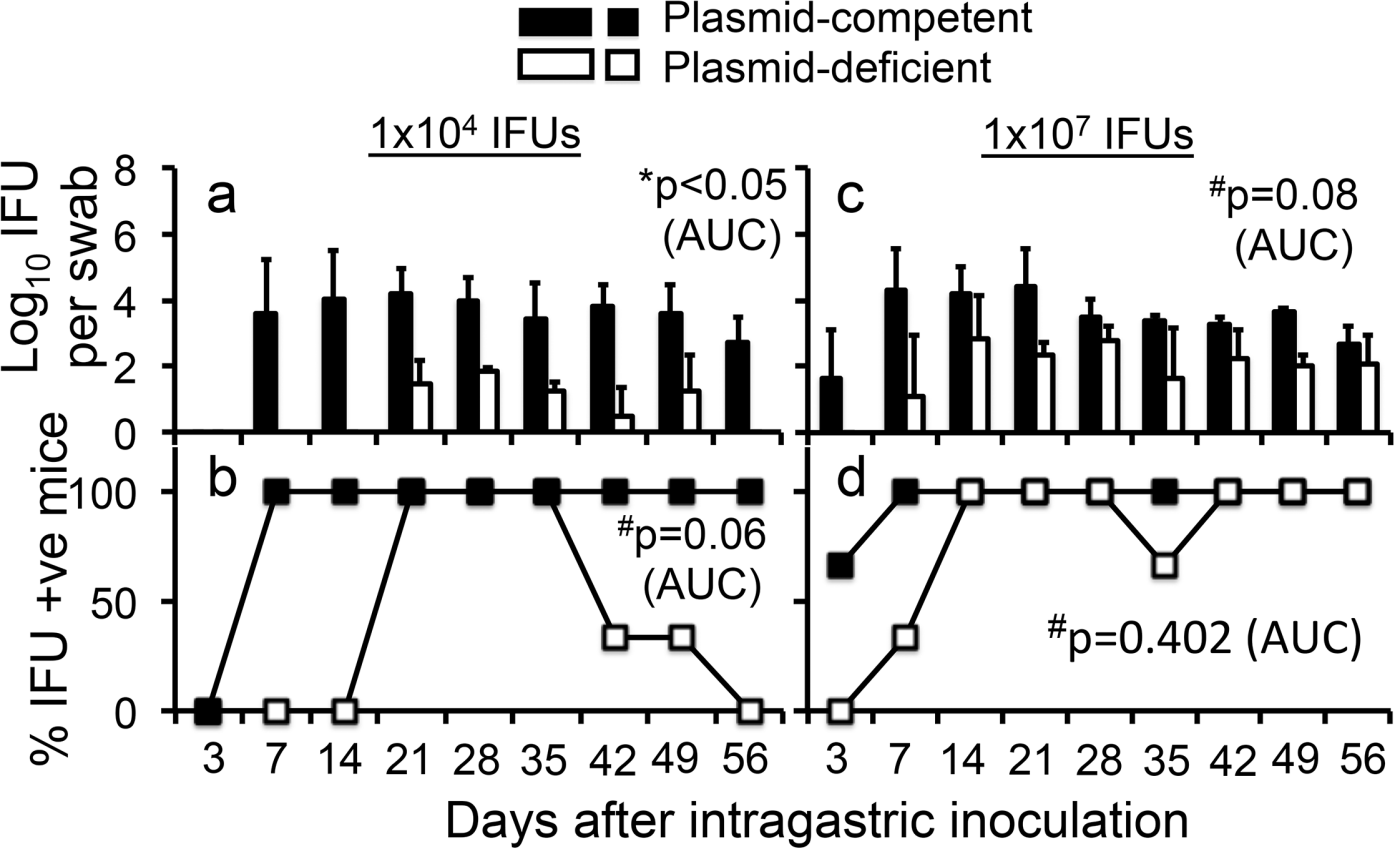
Comparing *C*. *muridarum* with or without plasmid for their ability to colonize the GI tracts of C57BL/6J mice following intragastric inoculation. The plasmid-competent (solid bar) or -deficient (open bar) *C*. *muridarum* organisms were inoculated intragastrically into female C57BL/6J mice with 1 x 10^4^ IFUs per mouse (panels a, n = 4, & b, n = 4) or 1 x 10^7^ IFUs (c, n = 3, & d, n = 3). At various time points post inoculation as indicated along the X-axis, rectal swabs were taken for titrating *C*. *muridarum* live organisms. The recovered live organisms were expressed as Log_10_ IFUs per swab (a & c) as displayed along the Y-axis and % of mice detected positive for IFUs at a given time point were plotted in panels b & d. The data were from two independent experiments. Note that plasmid-deficient *C*. *muridarum* developed significantly delayed/reduced shedding courses from the mouse GI tracts at the inoculation dose of 1 x 10^4^ IFUs (panel a, *p<0.05, b, #p = 0.06, both area under the curve, Wilcoxon rank sum) or 1 x 10^7^ IFUs (panel c, ^#^p = 0.08, d, p = 0.402, both area under the curve, Wilcoxon rank sum); # the lack of significance is probably caused by the limited sample sizes.

The above observations that blood levels of both plasmid-deficient and plasmid-competent *C*. *muridarum* genomes were similar but the plasmid-deficient strain displayed significantly reduced live organism shedding from the GI tract suggest a critical role of the plasmid in *C*. *muridarum* colonization of the GI tract. To test this hypothesis, we compared plasmid-competent and -deficient *C*. *muridarum* for their ability to colonize the mouse GI tract following an intragastric inoculation (Fig 4). When female C57BL/6J mice were intragastrically inoculated with 1 x 10^4^ IFUs per mouse, plasmid-competent *C*. *muridarum* established GI colonization in 100% of the mice by day 7 post inoculation and maintained a steady level of colonization thereafter, which is consistent with what we have previously shown [51]. However, plasmid-deficient *C*. *muridarum* failed to stably colonize the mouse GI tract at the same inoculation dose. Live plasmid-deficient *C*. *muridarum* organisms were only detectable by day 21 and the titers were significantly lower (Fig 4A). Furthermore, colonization by the plasmid-deficient *C*. *muridarum* organisms was transient since mice started to clear the infection on day 42. By day 56, all mice completely cleared the plasmid-deficient *C*. *muridarum* organisms from the rectal swabs. When the plasmid-competent and -deficient *C*. *muridarum* organisms were compared at a high inoculation dose (1 x 10^7^ IFUs), the same trend of differences in shedding courses remained. The 1000-fold increase in inoculation dose with plasmid-competent *C*. *muridarum* did not increase the titer of live organisms recovered from the rectal swabs, which is consistent with what we previously reported [51]. However, the higher dose inoculation seemed to help the plasmid-deficient *C*. *muridarum* stabilize their colonization in the GI tract. Nevertheless, even at the saturating dose, the live organism shedding of plasmid-deficient *C*. *muridarum* in the rectal swabs was still somewhat delayed and reduced although without any statistical significance. However, the shedding of plasmid-deficient *C*. *muridarum* reached to the level similar to that of the plasmid-competent *C*. *muridarum* towards the end of the shedding courses, which is similar to the overall trend of the shedding courses monitored following intravaginal or intravenous inoculation. Since at the inoculation dose of 1 x 10^7^ IFUs, the rectal swab shedding course of plasmid-deficient *C*. *muridarum* was still not as robust as that of the plasmid-competent *C*. *muridarum* at an inoculation dose of 1 x 10^4^ IFUs, we can conclude that the plasmid-deficient *C*. *muridarum* organisms were significantly less capable of colonizing the GI tract than the plasmid-competent organisms.

## Discussion

Although *Chlamydia* has been detected in the GI tracts, neither the mechanisms by which nor the significance for which *Chlamydia* colonizes the gut are known. Here, we have compared *C*. *muridarum* dependence on the cryptic plasmid designated pMoPn/pCM, a known chlamydial pathogenic determinant in the genital tract, for colonizing the mouse genital versus GI tracts. Our data has demonstrated that the plasmid is more important for *C*. *muridarum* to colonize the GI than the genital tracts.

First, *C*. *muridarum* with or without plasmid developed similar levels of live organism shedding from the mouse lower genital tract following intravaginal inoculation (Figs 1A, 1B, 2A and 2B), suggesting that the plasmid is not essential for *C*. *muridarum* to colonize the lower genital tract. These findings are consistent with previous observations that plasmid-free *C*. *muridarum* developed as robust live organism shedding in the vaginal swabs as did plasmid-competent *C*. *muridarum*, although the plasmid deficiency significantly attenuated *C*. *muridarum* pathogenicity in the mouse upper genital tract [13, 16, 17, 27]. The isolation of plasmid-deficient *C*. *trachomatis* strains from human genital tracts [37, 38] and the failure of the plasmid to promote *C*. *trachomatis* colonization in the genital tracts of nonhuman primates [39] suggest that the plasmid may not be always essential for *C*. *trachomatis* colonization in the genital tracts. Second, plasmid deficiency significantly reduced the spreading of the genital tract *C*. *muridarum* to the GI tract (Figs 1C, 1D, 2C and 2D), suggesting a role of the plasmid in promoting chlamydial spreading to the GI tract. It is worth pointing out that the different efficiency in spreading to the GI tract between the plasmid-competent and -deficient *C*. *muridarum* might also reflect variable efficiencies in oral-fecal transmission between these two types of the organisms since mice from the same group were housed in the same cage. Mice housed in the same cage can orally take up each other’s excretions from the genital tracts. However, we have previously shown that the predominant route of spreading of the genital tract *C*. *muridarum* to the GI tract was via the blood circulation even when mice were housed in the same cage [47, 50]. In addition, the follow-up experiments in which *C*. *muridarum* organisms were delivered into mice intravenously or intragastrically will further reduce the concern. Third, *C*. *muridarum* with or without plasmid maintained similar levels of genome copies in the blood after intravenous injection (Fig 3A), suggesting that the reduced spreading of the plasmid-deficient *C*. *muridarum* to the GI tract is not due to lack of *C*. *muridarum* survival in the blood. However, similar levels of genomes do not necessarily mean similar levels of infectivity. If a higher portion of plasmid-deficient *C*. *muridarum* were inactivated in the blood, the plasmid-deficient *C*. *muridarum* organisms might end up with less colonization in the GI tract. The possibility was minimized by the following experiment. Fourth, blood-borne *C*. *muridarum* was significantly less efficient in colonizing the GI tract in the absence of the plasmid (Fig 3B), suggesting that the plasmid is required for hematogenous *C*. *muridarum* to colonize the GI tract. Finally, the colonization of the intragastrically inoculated *C*. *muridarum* in the GI tract was highly dependent on the plasmid (Fig 4). Thus, we have presented evidence demonstrating that the reduced spreading of the plasmid-deficient *C*. *muridarum* organisms from the genital to the GI tracts was due to their reduced ability to colonize the GI tract.

The next question is how the plasmid promotes chlamydial colonization in the GI tract. It is now known that among the 8 plasmid-encoded pGPs [29], pGP1, 2 & 6 are important for plasmid maintenance while pGP3, 4, 5, 7 & 8 are not [33–35]. pGP4 is a master positive regulator of both plasmid-encoded and chromosomal genes [33, 34] while pGP5 is a negative regulator [35]. pGP3, 7 or 8 have minimal impact on other gene expression [33–35]. pGP3 deficiency largely phenocopied plasmid-deficiency when infecting animal genital tracts [13, 32]. pGP3 is thus considered a key virulence factor for plasmid-dependent pathogenicity in the mouse upper genital tract. The question is whether pGP3 is also required for promoting *C*. *muridarum* colonization in the GI tract is under investigation. It is worth noting that the less efficient colonization of plasmid-deficient *C*. *muridarum* in the GI tract was more obvious at the beginning and by 8 weeks after the initial inoculation (regardless of the routes of inoculation), the plasmid-deficient *C*. *muridarum* managed to reach to the levels similar to those of plasmid-competent *C*. *muridarum*. The gap in live organism shedding between the plasmid-deficient and -competent organisms was gradually closed up, which was not caused by contamination of the plasmid-deficient *C*. *muridarum*-infected mice with plasmid-competent *C*. *muridarum* or re-acquisition of plasmid by the plasmid-deficient *C*. *muridarum* since the plasmid-deficient *C*. *muridarum* isolated from the rectal swabs collected at the late stage of the infection time course remained plasmid-free (data not shown). Our hypothesis is that this may reflect the adaptation of plasmid-deficient *C*. *muridarum* to the gut environment. Ongoing experiments are underway in investigating the mechanism of the adaptation.

Although *C*. *muridarum* organisms can readily infect extra-GI tract mucosal tissues [46], *C*. *muridarum* only achieves long-term colonization in the mouse GI tract [48–51]. The *C*. *muridarum* infection in extra-GI tract tissues is often quickly cleared, for example, in 4 to 5 weeks from the genital tract [6, 19, 20, 36, 55, 56] and 1 to 2 weeks from the airway [57, 58]. More importantly, the long-lasting *C*. *muridarum* colonization in the GI tract seems to be nonpathogenic while *C*. *muridarum* induces inflammatory pathologies in the extra-GI tract tissues, including hydrosalpinx in the genital tract [6, 19, 20, 36, 55, 56] and pneumonitis in the airway [57, 58]. In fact, *C*. *muridarum* used to be called mouse pneumonitis agent or MoPn [59]. This is why *C*. *muridarum* infection in the genital tract or airway but not the GI tract has been frequently used as disease models [4, 58, 60]. The above analyses suggest that *C*. *muridarum* may have fully adapted to the mouse GI tract as a result of its repeated oral-fecal route transmission. Although directly proving the oral-fecal route as a natural pathway for *C*. *muridarum* to spread between mice may require extensive field studies, the current study has provided a piece of novel biological evidence for supporting this hypothesis.

Last but not least, why should we study *C*. *muridarum* colonization in the mouse GI tract since *C*. *muridarum* is harmless in the GI tract? Although *C*. *muridarum* infection in the mouse genital tract has been used to study chlamydial pathogenesis due to its ability to induce upper genital tract pathologies and complications similar to those observed in *C*. *trachomatis*-infected women [1–3], the plasmid is not very important for *C*. *muridarum* to infect the mouse genital tract [13, 16, 17, 27, 36]. Since maintaining an extra-chromosomal plasmid must serve a biological purpose, the above observations suggest that *C*. *muridarum* is not naturally transmitted between mice via the genital tract. Instead, as discussed previously and supported by the novel finding from the current study, *C*. *muridarum* may have acquired the plasmid for adaption to the mouse GI tract during its natural transmission via the oral-fecal route. Consistently, *C*. *muridarum* plasmid (or other virulence factors) is more important for *C*. *muridarum* to colonize the GI tract than to infect the genital tract. Indeed, due to the lack of obvious deficiency in infecting the mouse genital tract by plasmid-free *C*. *muridarum*, it has been difficult to investigate the mechanisms by which the plasmid promotes *C*. *muridarum* induction of hydrosalpinx in the upper genital tract [28]. Fortunately, the significant deficiency in colonizing the GI tract by plasmid-deficient *C*. *muridarum* may have provided a useful platform (with a clear phenotype) for defining the host factors targeted by the plasmid-encoded or -regulated effectors. It is likely that the same mechanisms that *C*. *muridarum* has evolved for improving its fitness with the mouse GI tract mucosal tissues may be used by *C*. *muridarum* to infect the genital tract tissues when the organisms are artificially introduced to the genital tract, the latter of which often results in pathology that mimics those found in women with *C*. *trachomatis* infection in the genital tract. Thus, investigating the interactions of *C*. *muridarum* with the mouse GI tract may represent an unconventional but productive approach for understanding chlamydial pathogenesis in the genital tract. In addition, despite the fact that *C*. *trachomatis* is naturally transmitted sexually between humans while oral-fecal route transmission is less likely, *C*. *trachomatis* has been detected in the GI tracts of humans [42–45, 61] and can infect human enteroendocrine cells [62]. Women practicing oral/anal sex can introduce *C*. *trachomatis* into their GI tracts [43, 45]. However, there is no clear association of *C*. *trachomatis* (non-LGV serovars) with GI pathology in humans [63, 64], which is similar to *C*. *muridarum* colonization in the mouse GI tract. Although the medical significance of the GI tract *C*. *trachomatis* in humans remains unclear, it may be worth the effort to find out why and how *Chlamydia* manages to colonize the GI tract.
